# Taping patients with clinical signs of subacromial impingement syndrome: the design of a randomized controlled trial

**DOI:** 10.1186/1471-2474-12-188

**Published:** 2011-08-17

**Authors:** Joeri Kalter, Adri T Apeldoorn, Raymond W Ostelo, Nicholas Henschke, Dirk L Knol, Maurits W van Tulder

**Affiliations:** 1 Department of Epidemiology & Biostatistics, EMGO Institute for Health & Care Research, VU University Medical Centre, Amsterdam, the Netherlands; 2 Department of Epidemiology & Biostatistics, EMGO Institute for Health & Care Research, VU University Medical Centre, Amsterdam, the Netherlands & Department of Health Sciences, Faculty of Earth & Life Sciences, VU University Amsterdam, the Netherlands; 3 Department of Epidemiology & Biostatistics, EMGO Institute for Health & Care Research, VU University Medical Centre, Amsterdam, the Netherlands & The George Institute for Global Health, Sydney, Australia

## Abstract

**Background:**

Shoulder problems are a common complaint of the musculoskeletal system. Physical therapists treat these patients with different modalities such as exercise, massage, and shoulder taping. Although different techniques have been described, the effectiveness of taping has not yet been established. The aim of this study is to assess the effectiveness and cost-effectiveness of usual physical therapy care in combination with a particular tape technique for subacromial impingement syndrome of the shoulder compared to usual physical therapy care without this tape technique in a primary healthcare setting.

**Methods and design:**

An economic evaluation alongside a randomized controlled trial will be conducted. A sample of 140 patients between 18 and 65 years of age with a diagnosis of subacromial impingement syndrome (SAIS) as assessed by physical therapists will be recruited. Eligible patients will be randomized to either the intervention group (usual care in combination with the particular tape technique) or the control group (usual care without this tape technique). In both groups, usual care will consist of individualized physical therapy care. The primary outcomes will be shoulder-specific function (the Simple Shoulder Test) and pain severity (11-point numerical rating scale). The economic evaluation will be performed using a societal perspective. All relevant costs will be registered using cost diaries. Utilities (Quality Adjusted Life Years) will be measured using the EuroQol. The data will be collected at baseline, and 4, 12, and 26 weeks follow-up.

**Discussion:**

This pragmatic study will provide information about the effectiveness and cost-effectiveness of taping in patients presenting with clinical signs of SAIS.

**Trial registration:**

Trial registration number: NTR2575

## Background

Shoulder problems are a common complaint of the musculoskeletal system. It is the second most common musculoskeletal disorder following low back pain [1] and results in high morbidity [2]. Of all shoulder problems, subacromial impingement syndrome (SAIS) is the most frequent diagnosis [3], accounting for 44-65% of all shoulder problems [4-6]. In general, SAIS (also known as external impingement) is defined as the entrapment of the rotator cuff and subacromial bursa between the humerus and the coracoacromial arch [7].

Patients with SAIS are commonly treated by a physical therapist (PT). Various therapeutic options have been described, however, most are lacking a rigorous scientific background and there is uncertainty about the associated costs [8]. Some reviews have reported that exercise and manual treatment are most effective in relieving pain and improving function in SAIS, but the evidence for effectiveness is still under debate [8,9].

A promising modality in physical therapy is the application of taping. The essential function of the tape is to provide support during movement. However, research indicates that the tape also plays a role in decreasing the upper trapezius muscle activity [10] and offers a constant input on the proprioceptive system of the upper body muscles which support the active movement [11].

Three studies have examined the clinical effects of taping patients with SAIS using leukotape in combination with fixomull stretch (BSN medical^®^) [12-14]. In a pilot study of 22 patients with unilateral shoulder pain of more than six weeks, Miller et al. [12] compared taping as an adjunct to usual physical therapy with usual physical therapy alone. They found a strong trend toward reduced pain and improved function in favor of scapular taping at two weeks. The magnitude of these differences was reduced at a follow-up at six weeks. Case studies of taping patients with SAIS have been published by Host et al. [13] and Shamus and Shamus [14]. Both studies found a reduction in pain and an improvement of function. In sum, the effects in pain relief and improving shoulder function due to shoulder taping looks promising. However, study samples were small or had no control group. In addition, tape techniques vary and we must conclude that the evidence for its usefulness is still limited. Therefore, the primary aim of this study is to assess the effectiveness and cost-effectiveness of usual physical therapy care in combination with one particular tape technique for subacromial impingement syndrome of the shoulder compared to usual physical therapy care alone in a primary health care setting.

## Methods/Design

### Design

**Figure 1:**
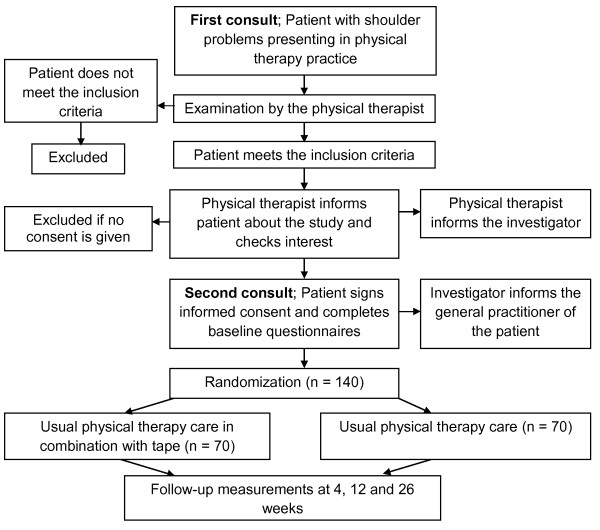
**Design of the study**.

An economic evaluation will be conducted alongside a randomized controlled trial (RCT). Figure 1 provides an overview of the study design. Outcomes will be measured at baseline, 4, 12 and 26 weeks follow-up.

### Setting

A sample of 140 patients between 18 and 65 years of age with clinical signs of SAIS will be recruited from the primary physical therapy setting in the Netherlands between September 2010 and November 2011.

### Ethical approval

The Medical Ethics Committee of the VU University Medical Centre in Amsterdam has approved the study protocol (registration number: 2010\119).

### Study population

To be eligible for the trial, patients must meet the following criteria as assessed by the participating PTs; Two positive impingement tests indicating SAIS (see section impingement tests), age between 18 and 65 years, and accepting the consequences of participation in the study. Patients will be excluded if they have a primary SAIS (anatomical abnormalities as scapulohumeral joint dysplasia, total cuff tear etc. confirmed with radiography and/or diagnostic ultrasound with exception of calcifications, minor arthrosis, and partial cuff tears]), been operated previously at the shoulder or cervical spine, a rheumatic disease such as polymyalgia rheumatica, rheumatoid arthritis, lupus erythematosis and fibromyalgia, a severe arthritis of the glenohumeral joint, had three or more subacromial corticosteroid injections in the last year, a (suspected) severe disease such as malignancy, had severe trauma of the shoulder in the last 6 months, a neurologic disease with negative consequences for the shoulder (such as cerebral vascular accident, multiple sclerosis, Parkinson's disease), type II diabetes, had a luxation or fracture of the affected shoulder, a cervico-radicular syndrome, a pathology of organs with negative consequences for the shoulder, dementia, a psychiatric disease, an insufficient understanding of the Dutch language, a bad condition of the skin around the shoulder because of a skin disease, and an allergy to tape.

### Recruitment of patients

At the first consult, PTs will inform all eligible patients who attend their primary care clinic about the study and will assess patients for the in- and exclusion criteria. Patients will be further instructed about the study through a patient information letter. For patients interested in participating, the PT will provide contact details of the patients to the investigators. One of the investigators will phone the patient before the second consult to answer potential questions and to provide information. If the patient decides to participate, they will be asked to fill in baseline questionnaires (digital or paper) before the second physical therapy consult. At the second consult, the patient will sign an informed consent form. Thereafter, the PT will open a numbered opaque sealed envelope containing the treatment allocation (i.e. usual physical therapy care in combination with one particular tape technique or usual physical therapy care without this tape technique).

### Treatment allocation

Prior to the study, every practice will receive 10 to 20 concealed envelopes, which contain an allocation to one of the two treatment strategies. The randomization process will be generated by computer. To prevent unequal treatment-group sizes, the patients will be randomized according to a stratified block randomization method, in blocks of four. The PTs will not know the sequence of the treatment allocation codes of each practice to guarantee allocation concealment.

### Impingement tests

Many different diagnostic criteria for the SAIS have been suggested [15]. In general, clinicians base the diagnosis of SAIS on the history of the patient and specific signs and symptoms. Research findings suggest that single tests have limited use in informing diagnosis and a combination of tests improves clinical utility [9,15]. For the present study, two or more positive well-known SAIS tests will indicate a SAIS, which is in concordance with previous pragmatic studies [16]. The following SAIS tests will be used: the painful arc of abduction test [17], the empty can test (Jobe test) [18], the external rotation resistance test [19] and the Hawkins-Kennedy test [20]. The reliability of all tests is acceptable for clinical use [15].

### Physical therapists

All patients will be treated by PTs of the participating clinics. Prior to the study, all PTs will follow a 1½-hour training session in which the rationale of the study will be described and discussed, and the taping method will be instructed and trained. The PTs will receive a detailed manual of the study protocol including a standardized eligibility checklist, and a description of the impingement tests and the taping technique.

### Tape method

**Figure 2:**
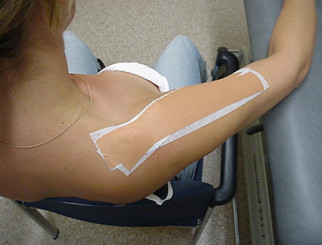
**The tape is applied to the shoulder in 80° abduction and 30° forward flexion**.

**Figure 3:**
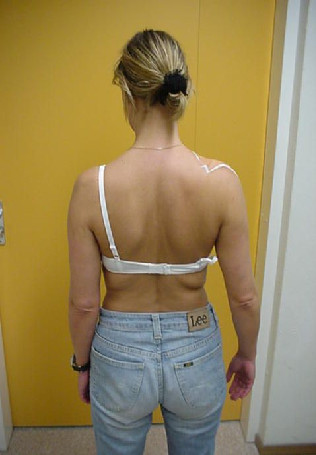
**In a relaxed position, the arm hangs in slight abduction due to the tension of the tape**.

Patients who are allocated to usual physical therapy in combination with tape will be treated at the end of each treatment session for at least 4 weeks by a modified tape technique after Shamus and Shamus [14]. In this technique, the arm of the patient lays on the treatment table, with the shoulder in 80° abduction in the plane of the scapula (Figure 2). Two strips of rigid tape (leukotape, BSN medical^®^) will be placed upon two strips of elastic tape (fixomull stretch, BSN medical^®^) without tension. When the arm of the patient is brought back to a relaxed position along the body, the arm will be slightly abducted from the body (approximately 10°) due to the tension of the tape (Figure 3). The tape will be worn for a minimum of 2 and a maximum of 7 days. Tape will be worn for short periods (2-3 days) for patients with severe pain and at the beginning of the tape period, and longer for patients with less pain and who are familiar with wearing the tape. In addition, patients with severe pain will be taped with the shoulder in less abduction (50-70°) so that the tape produces less tension when the arm is in the relaxed position. At least one to two days before the next treatment, the tape will be removed by the patient in order to let the skin recover from the tape. The PT is free to determine the abduction angle of the shoulder in which the shoulder will be taped, the exact days the tape will be worn and the number of treatments per week in order to reflect clinical practice. During the first 4 weeks, the treatment with tape will be stopped if the patient is free of pain, allergic to tape or reports a substantial increase in complaints because of the tape.

### Usual care

Usual care will consist of individualized tailored physical therapy. The content of treatment, i.e. the choice of interventions, is at the discretion of the PT. In general, therapy is expected to include exercises to improve mobility and muscle function, passive mobilizations, instructions (posture and movement), massages and ice packages [8]. Furthermore, if the patient is not allocated to usual care in combination with shoulder taping, the use of kinesiotape will be allowed. In the Netherlands, kinesiotape is frequently used, but the theoretical concept, the tape structure, and the techniques are not comparable to the tape and tape technique used in this study [21,22]. During the first four weeks the patients are requested not to receive any co-interventions (such as complementary and/or alternative medicine [CAM], injection therapy or surgery). The use of (pain) medication is allowed in both groups.

For both interventions the number of treatment sessions, the treatment modalities, the treatment goals, any deviations from the protocol, and reasons for prematurely terminating treatment (e.g. skin problems due to the tape) will be recorded. In addition, PTs in both groups familiar with the classification system described in Cools and Walravens [23] will classify the patients according to this system, in order to identify subgroups that respond best to the tape technique used. The principal investigators will regularly monitor the PTs for compliance with the treatment protocols, by means of phone calls and e-mail contact.

### Data collection

At baseline, patients will be asked to fill in digital or paper questionnaires before the start of the treatment, and after 4 and 26 weeks follow-up. After 12 weeks, patients will be called by the investigators to collect data about global perceived recovery, pain severity, and costs (Table 1).

**Table 1:** Overview of data collection

Primary outcomes		
Shoulder-specific function	Simple Shoulder Test	Baseline, 4 and 26 weeks
Pain severity	11-point numerical rating scale	Baseline, 4, 12 and 26 weeks

**Secondary outcomes**		

Global perceived recovery	7-point Likert scale	4, 12 and 26 weeks
Primary functional problem	11-point numerical rating scale	Baseline, 4 and 26 weeks

**Economic evaluation**		

Costs	Cost diary	4, 12 and 26 weeks
Health related quality of life	EuroQol	Baseline, 4 and 26 weeks

**Other**		

Demographic data	Questionnaire	Baseline
General health	Short-form 36	Baseline

The baseline questionnaire will include questions about socio-demographic characteristics (e.g. age, gender, and marital status), pain severity, shoulder-specific function, primary functional problem, health related quality of life and general health. General health will be evaluated with the Short-Form 36 (SF-36) [24]. This questionnaire consists of 36 questions that can be aggregated to form eight subscales (physical functioning, mental health, general health perceptions, pain, role limitations physical, role limitations emotional, social functioning, and vitality) and two sum scales (physical and mental component scales). The scores on all subscales range from 0-100, with higher scores indicating better outcomes. It is a widely used measurement instrument with satisfactory validity, reproducibility and responsiveness to change [25]. The Dutch translation has been found to be sufficiently valid [26].

### Outcome measures

The primary outcome measures will be:

**The Simple Shoulder Test (SST) **[27] measuring shoulder function. The SST will measure gains and losses of function over time through a series of 12 questions with dichotomous "yes" or "no" response options. More scores on yes relates to less pain and less disability of the shoulder. The SST has been found to be sufficiently valid, reliable and responsive [28,29].

**The numerical rating scale (NRS) **measuring pain severity. The NRS (0 = no pain to 10 = worst imaginable pain) is well-accepted in clinical research with good validity and reliability [30-32].

Secondary outcome measures include:

**Global perceived recovery**, measured by self-assessment on a 7-point Likert scale ranging from "completely recovered to "worse than ever" [33]. This will be dichotomized into success (complete and much recovered) and non-success (slightly recovered, no change, slightly worse, much worse, and worse than ever).

**Perceived recovery from a primary functional problem**, measured by an 11-point NRS. Prior to the treatment programme, the patients will select one functional activity that often occurs and cannot be avoided. The patient will be asked to describe the activity as specific as possible and wherever possible to quantify the activity in terms of time, distance, weight lifted, and so on. Beurskens et al. investigated the value of this instrument in patients with low back pain, but used a more complex procedure compared to our pragmatic approach [34]. They concluded that the responsiveness is good and changes in function problems were displayed properly.

### Blinding

The treating PTs and patients cannot be blinded for the treatment allocation because of the knowledge of receiving the modality. The investigators, who will conduct the data collection and data entry, will not be blinded. However, almost all outcome measures will be collected using questionnaires without personal contact with the patient. The participating statistician will perform the analyses blinded for treatment allocation.

### Sample size

Power calculations, based on the studies carried out by Santamato et al. [35] and Tashjian et al. [36] were performed for the 2 main outcomes (power 0.95; alpha 0.05). To detect a clinically relevant mean difference between the two treatment groups of 2 points on the SST [36] (standard deviation [SD] 2), 26 patients are needed per group. To detect a clinically relevant mean difference of 2 points (SD 3) for pain (11-point NRS) [35], 2 groups of 59 patients are needed. Anticipating a potential drop-out of 15%, 70 participants per treatment group (total n = 140) will be recruited.

### Data-analysis

Baseline characteristics of the two treatment groups will be compared regarding the main prognostic characteristics. The primary analysis will be on an intention-to-treat basis. The data will be analyzed in a linear mixed model with measurements at baseline, 4, 12 and 26 weeks. In this model, the effect of the interaction is time by treatment interaction. The included levels will be; repeated measures (i.e. time), patient, PT, and PT practice. The treatment effects between baseline measurement and follow-up will be calculated between the two treatment groups with a 95% confidence interval. If prognostic factors are unevenly distributed, multivariate analysis will be used to correct for the differences between the groups.

The influence of deviations on the treatment protocol will be evaluated using a per protocol analysis. A deviation of the protocol is defined as not receiving treatment after allocation, withdrawal from therapy after one to three visits and not being treated according to patient's treatment allocation.

### Economic evaluation from a societal perspective

The economic evaluation will be conducted from a societal perspective. Both a cost-effectiveness and cost-utility analysis will be performed. Shoulder function (SST) and pain severity (NRS) will be primary outcomes in the cost-effectiveness analysis. Health-related quality of life will be measured with the EuroQol (EQ-5D) [37]. This questionnaire assesses 5 dimensions (mobility, self-care, usual activities, pain/discomfort and anxiety/depression) on a 3-point scale; no problems, moderate problems and severe problems. The questionnaire is appropriate for estimating quality-adjusted life-years (QALYs), and can be used for cost-utility analysis. The total score is expressed in utilities according to the Dolan model [38]. QALYs will be calculated by multiplying the utility of a health state by the time spent in this health state, based on the Dutch valuation tariff [39].

Direct and indirect costs will be measured by means of self-completed cost diaries [40]. The following costs will be evaluated: I) health care costs (e.g. primary care, medical specialist care, prescription of medication, and hospitalization); II) patient costs (out-of-pocket expenses, such as over-the-counter medication and CAM); and III) costs of loss of productivity (work absenteeism).

Costs will be valued according to the guidelines published in the updated handbook for economic evaluation in the Netherlands [41]. For paid labour, the costs will be calculated, using both the human capital approach and the friction cost approach. The incremental costs from the intervention group over the usual care group will be compared to incremental outcomes. The incremental cost-effectiveness ratios (ICER) will be calculated with the mean differences in costs and effects. To calculate the mean differences in costs and to analyse the uncertainty around the ICERs, the bias-corrected percentile bootstrapping method (5,000 replications) will be used. The uncertainties around the ICERs will be presented in a cost-effectiveness plane. Finally, the probability that usual care with the particular tape technique is cost-effective in comparison to usual care for various thresholds decision makers are willing to pay to gain one extra unit of effect will be presented on a cost-effectiveness acceptability curve.

## Discussion

Few studies have been performed evaluating the clinical effects of taping shoulders in patients presenting clinical signs and symptoms of SAIS, although some promising results have been reported [12-14]. It is interesting to note that there have been some studies for other patient populations using leukotape in combination with fixomull stretch. Peterson [42] described a taping technique with electrical stimulation in one patient with central cord syndrome and bilateral shoulder subluxation and concluded that the shoulder taping along with electrical stimulation and a rehabilitation program may have played a role in the reduction of the patient's shoulder subluxation. In a RCT, the effect of shoulder taping in 98 patients with a stroke was studied [43]. They found a non significant trend for less pain and better function in strapped patients after 6 weeks. Finally, a number of authors have investigated the effect of shoulder taping on electro-activity of shoulder muscles, but their findings are contradictory [10,11,44,45].

In sum, the use of shoulder taping is a promising modality in improving outcomes on pain relief and function improvement in those presenting to primary physical therapy care with signs and symptoms of SAIS, however the method has not been adequately tested.

To our knowledge this is the first RCT examining the possible additional effect of taping using a modified technique according to Shamus and Shamus [14] compared to usual physical therapy alone in primary care patients presenting with signs and symptoms of SAIS. In addition, it includes the first economic evaluation on taping for SAIS.

## Competing interests

Leukotape P combi packs (patella taping kits) will be provided by BSN medical^®^. The funder will have no role in the design, conduct, analysis or writing of the report.

## Authors' contributions

All authors participated in the design of the study. JK drafted the manuscript with input from the other authors. All authors read, revised and approved the final manuscript.

## Pre-publication history

The pre-publication history for this paper can be accessed here:

http://www.biomedcentral.com/1471-2474/12/188/prepub

## References

[B1] Picavet HS Schouten JS Musculoskeletal pain in the Netherlands: prevalences, consequences and risk groups, the DMC(3)-study Pain 2003 102 1-2 167 178 10.1016/s0304-3959(02)00372-x 12620608

[B2] Ostor AJ Richards CA Prevost AT Speed CA Hazleman BL Diagnosis and relation to general health of shoulder disorders presenting to primary care Rheumatology 2005 44 6 800 805 10.1093/rheumatology/keh598 15769790

[B3] Michener LA McClure PW Karduna AR Anatomical and biomechanical mechanisms of subacromial impingement syndrome Clin Biomech 2003 18 5 369 379 10.1016/S0268-0033(03)00047-0 12763431

[B4] Vecchio P Kavanagh R Hazleman BL King RH Shoulder pain in a community-based rheumatology clinic Br J Rheumatol 1995 34 5 440 442 10.1093/rheumatology/34.5.440 7788173

[B5] Van der Windt DA Koes BW Boeke AJ Deville W De Jong BA Bouter LM Shoulder disorders in general practice: prognostic indicators of outcome Br J Gen Pract 1996 46 410 519 523 8917870 PMC1239746

[B6] Van der Windt DA Koes BW De Jong BA Bouter LM Shoulder disorders in general practice: incidence, patient characteristics, and management Ann Rheum Dis 1995 54 12 959 964 10.1136/ard.54.12.959 8546527 PMC1010060

[B7] Flatow EL Soslowsky LJ Ticker JB Pawluk RJ Hepler M Ark J Mow VC Bigliani LU Excursion of the rotator cuff under the acromion. Patterns of subacromial contact Am J Sports Med 1994 22 6 779 788 10.1177/036354659402200609 7856802

[B8] Michener LA Walsworth MK Burnet EN Effectiveness of rehabilitation for patients with subacromial impingement syndrome: a systematic review J Hand Ther 2004 17 2 152 164 10.1197/j.jht.2004.02.004 15162102

[B9] Kelly SM Wrightson PA Meads CA Clinical outcomes of exercise in the management of subacromial impingement syndrome: a systematic review Clin Rehabil 2010 24 2 99 109 10.1177/0269215509342336 20103573

[B10] Smith M Sparkes V Busse M Enright S Upper and lower trapezius muscle activity in subjects with subacromial impingement symptoms: is there imbalance and can taping change it? Phys Ther Sport 2009 10 45 50 10.1016/j.ptsp.2008.12.002 19376471

[B11] Alexander CM Stynes S Thomas A Lewis J Harrison PJ Does tape facilitate or inhibit the lower fibres of trapezius? Man Ther 2003 8 1 37 41 10.1054/math.2002.0485 12586560

[B12] Miller P Osmotherly P Does scapula taping facilitate recovery for shoulder impingement symptoms? A pilot randomized controlled trial J Man Manip Ther 2009 17 1 E6 E13 10.1179/106698109790818223 20046559 PMC2704341

[B13] Host HH Scapular taping in the treatment of anterior shoulder impingement Phys Ther 1995 75 9 803 812 7659740 10.1093/ptj/75.9.803

[B14] Shamus JL Shamus EC A taping technique for the treatment of acromioclavicular joint sprains: a case study J Orthop Sports Phys Ther 1997 25 6 390 394 9168346 10.2519/jospt.1997.25.6.390

[B15] Michener LA Walsworth MK Doukas WC Murphy KP Reliability and diagnostic accuracy of 5 physical examination tests and combination of tests for subacromial impingement Arch Phys Med Rehabil 2009 90 11 1898 1903 10.1016/j.apmr.2009.05.015 19887215

[B16] Kromer TO De Bie RA Bastiaenen CH Effectiveness of individualized physiotherapy on pain and functioning compared to a standard exercise protocol in patients presenting with clinical signs of subacromial impingement syndrome. A randomized controlled trial BMC Musculoskelet Disord 2010 11 114 10.1186/1471-2474-11-114 20534140 PMC2889850

[B17] Kessel L Watson M The painful arc syndrome. Clinical classification as a guide to management J Bone Joint Surg Br 1977 59 2 166 172 873977 10.1302/0301-620X.59B2.873977

[B18] Jobe FW Moynes DR Delineation of diagnostic criteria and a rehabilitation program for rotator cuff injuries Am J Sports Med 1982 10 6 336 339 10.1177/036354658201000602 7180952

[B19] Park HB Yokota A Gill HS El Rassi G McFarland EG Diagnostic accuracy of clinical tests for the different degrees of subacromial impingement syndrome J Bone Joint Surg Am 2005 87 7 1446 1455 10.2106/JBJS.D.02335 15995110

[B20] Hawkins RJ Kennedy JC Impingement syndrome in athletes Am J Sports Med 1980 8 3 151 158 10.1177/036354658000800302 7377445

[B21] Hsu YH Chen WY Lin HC Wang WT Shih YF The effects of taping on scapular kinematics and muscle performance in baseball players with shoulder impingement syndrome J Electromyogr Kinesiol 2009 19 6 1092 1099 10.1016/j.jelekin.2008.11.003 19147374

[B22] Thelen MD Dauber JA Stoneman PD The clinical efficacy of kinesio tape for shoulder pain: a randomized, double-blinded, clinical trial J Orthop Sports Phys Ther 2008 38 7 389 395 18591761 10.2519/jospt.2008.2791

[B23] Cools AM Walravens M Oefentherapie bij schouderaandoeningen [Exercise therapy for shoulder disorders] 2007 Antwerpen: Standaard Uitgeverij

[B24] McHorney CA Ware JE Jr Raczek AE The MOS 36-Item Short-Form Health Survey (SF-36): II. Psychometric and clinical tests of validity in measuring physical and mental health constructs Med Care 1993 31 3 247 263 10.1097/00005650-199303000-00006 8450681

[B25] Wittink H Turk DC Carr DB Sukiennik A Rogers W Comparison of the redundancy, reliability, and responsiveness to change among SF-36, Oswestry Disability Index, and Multidimensional Pain Inventory Clin J Pain 2004 20 3 133 142 10.1097/00002508-200405000-00002 15100588

[B26] Van der Zee K Sanderman R Heyink J De psychometrische kwaliteiten van de MOS 36-item Short Form Health Survey (SF-36) in een Nederlandse populatie [The psychometric properties of the SF-36 in a Dutch population] Tijdschr Soc Gez 1993 71 173 191

[B27] Lippit SB Harryman DT Matsen FA Matsen FA, Fu FH, Hawkins RJ A practical tool for evaluation of function: the Simple Shoulder Test The shoulder: a balance of mobility and stability 1993 Rosemond (IL): American Academy of Orthopaedic Surgeons 501 518

[B28] Godfrey J Hamman R Lowenstein S Briggs K Kocher M Reliability, validity, and responsiveness of the simple shoulder test: psychometric properties by age and injury type J Shoulder Elbow Surg 2007 16 3 260 267 10.1016/j.jse.2006.07.003 17188906

[B29] Roy JS MacDermid JC Woodhouse LJ Measuring shoulder function: a systematic review of four questionnaires Arthritis Rheum 2009 61 5 623 632 10.1002/art.24396 19405008

[B30] Revill SI Robinson JO Rosen M Hogg MI The reliability of a linear analogue for evaluating pain Anaesthesia 1976 31 9 1191 1198 10.1111/j.1365-2044.1976.tb11971.x 1015603

[B31] Carlsson AM Assessment of chronic pain. I. Aspects of the reliability and validity of the visual analogue scale Pain 1983 16 1 87 101 10.1016/0304-3959(83)90088-X 6602967

[B32] Sriwatanakul K Kelvie W Lasagna L Calimlim JF Weis OF Mehta G Studies with different types of visual analog scales for measurement of pain Clin Pharmacol Ther 1983 34 2 234 239 10.1038/clpt.1983.159 6872418

[B33] Feinstein AR Clinimetrics 1987 New Have and London: Yale University Press

[B34] Beurskens AJ De Vet HC Koke AJ Lindeman E Van der Heijden GJ Regtop W Knipschild PG A patient-specific approach for measuring functional status in low back pain J Manipulative Physiol Ther 1999 22 3 144 148 10.1016/S0161-4754(99)70127-2 10220712

[B35] Santamato A Solfrizzi V Panza F Tondi G Frisardi V Leggin BG Ranieri M Fiore P Short-term effects of high-intensity laser therapy versus ultrasound therapy in the treatment of people with subacromial impingement syndrome: a randomized clinical trial Phys Ther 2009 89 7 643 652 10.2522/ptj.20080139 19482902

[B36] Tashjian RZ Deloach J Green A Porucznik CA Powell AP Minimal clinically important differences in ASES and simple shoulder test scores after nonoperative treatment of rotator cuff disease J Bone Joint Surg Am 2010 92 2 296 303 10.2106/JBJS.H.01296 20124055

[B37] EuroQol--a new facility for the measurement of health-related quality of life. The EuroQol Group Health Policy 1990 16 3 199 208 10109801 10.1016/0168-8510(90)90421-9

[B38] Dolan P Modeling valuations for EuroQol health states Med Care 1997 35 11 1095 1108 10.1097/00005650-199711000-00002 9366889

[B39] Lamers LM Stalmeier PF McDonnell J Krabbe PF Van Busschbach JJ Kwaliteit van leven meten in economische evaluaties: het Nederlands EQ-5D-tarief [Measuring the quality of life in economic evaluations: the Dutch EQ-5D tariff] Ned Tijdschr Geneeskd 2005 149 1574 1578 16038162

[B40] Goossens ME Rutten-Van Molken MP Vlaeyen JW Van der Linden SM The cost diary: a method to measure direct and indirect costs in cost-effectiveness research J Clin Epidemiol 2000 53 7 688 695 10.1016/S0895-4356(99)00177-8 10941945

[B41] Hakkaart-Van Roijen L Tan SS Bouwmans CAM Handleiding voor kostenonderzoek: Methoden en standaard kostprijzen voor economische evaluaties in de gezondheidszorg, geactualiseerde versie [Manual for costing: Methods and standard costs for economic evaluations in health care, updated version] 2010 Diemen: College voor zorgverzekeringen

[B42] Peterson C The use of electrical stimulation and taping to address shoulder subluxation for a patient with central cord syndrome Phys Ther 2004 84 634 643 15225082

[B43] Hanger HC Whitewood P Brown G Ball MC Harper J Cox R Sainsbury R A randomized controlled trial of strapping to prevent post-stroke shoulder pain Clin Rehabil 2000 14 4 370 380 10.1191/0269215500cr339oa 10945421

[B44] Cools AM Witvrouw EE Danneels LA Cambier DC Does taping influence electromyographic muscle activity in the scapular rotators in healthy shoulders? Man Ther 2002 7 3 154 162 10.1054/math.2002.0464 12372312

[B45] Selkowitz DM Chaney C Stuckey SJ Vlad G The effects of scapular taping on the surface electromyographic signal amplitude of shoulder girdle muscles during upper extremity elevation in individuals with suspected shoulder impingement syndrome J Orthop Sports Phys Ther 2007 37 11 694 702 18057671 10.2519/jospt.2007.2467

